# On Herps Tonsurans

**Published:** 1855-07

**Authors:** 


On Herpes Tonsurans (*Cazenave*). By Prof. Hebra. (Zeitschrift der K. K. Gessellschaft der Aertze zu Wien, Dec., 1854.)

Prof. Hebra, in this paper, gives first an historical account of the disease which, under the various names of porrigo scutulata, ringworm, teigne tondante, tinea tondens, tricophyton tonsurans, rhizophyto-alopecia, has been the subject of so much discussion.

Willan described the disease correctly, except that he derived it from little ichorous pustules, an unusual commencement. Plumbe gives a very good account of it. Alibert, apparently, was little acquainted with it; and Rayer, who gave it the name of teigne annulaire, erroneously considered it, with Willan, as a pustular affection. Later authors, as Green, Gibert, Riecke, Wilson, Fuchs, have described the disease either as favus, or as a species of herpes. In 1840, Cazenave described the disease under the term herpes tonsurans, although he knew it had been called porrigo scutulata by Willan, and teigne tondante by his countryman Mahon. In 1845, Malmsten discovered a fungus in the roots of the hair, and Gruby soon afterwards confirmed the observation. Very lately, Robin and Bazin have also carefully described the cryptogamic plant, though the plate given by Robin does not (according to Hebra) represent the fungus of herpes tonsurans, but that of plica polonica.

Hebra then describes the *symptoms* as follows:

1. On hairless parts there are two forms.

(*a*) Vesicles with clear or sometimes yellow contents, grouped or isolated, or normally-colored on reddened patches of skin. Except in paucity of number, these vesicles are not different from those of herpes preputialis, labialis, etc. In a few hours, however, they dry and form a thin, yellow-brown scurf, which is sometimes surrounded by a ring of vesicles, which rapidly run through the same course. The vesicular character is so decided, that Hebra thinks Cazenave quite justified in the name he has adopted.

(*b*) More frequently than the vesicular form of the herpes tonsurans is the second or macular form: small, deep-red spots, elevated in the very slightest degree above the level of the skin, form and become covered with thin, white scales. In a few days, the increase of the spots at their border has augmented the size of the patch to that of a sixpence, the outer border being more defined and redder than the center. Afterwards the center loses its clear, red-color, and becomes bluish red, or yellow, or even natural. This appearance characterizes the ringworm of the English. Frequently many maculæ begin at once; they pass and fuse each other, and give rise to various shapes.

2. On hairy parts the herpes tonsurans is chiefly distinguished from the disease on hairless parts by the dry, ragged remains of hairs of unequal length, as if the hairs had been cut off unequally; the hairs also often drop off, and the yellow white, or yellowish-brown, paper-thin, dry, bran-like scales of cuticle are disclosed.

The *diagnosis* of the disease on the hairy parts is especially given by the condition of the hair; on hairless parts, the number, arrangement, and figure of the vesicles, or the color, form, size of the spots, and the grouping of the patches, with the microscopic characters, suffice for the determination.

The microscopic examination of the cuticle, or of the roots of the hairs, or in the hairs, discloses, with a little care, the fungus, which is the smallest at present known.

The *cause* of this disease is unhesitatingly referred by Hebra to the growth of a fungus finding a fit soil. We have, therefore, only to learn how and in what manner the soil is prepared for the fungus, and how the fungus gets there. With respect to the first point, Hebra points out that a *macerated* cuticle represents the fit receptacle; and if in any way the cuticle becomes moistened, the fungus will grow, if it can arrive at the part. He calls particular attention to the fact that when fomentations are continually applied to limbs, an eruption of little vesicles or maculæ often appears; in many cases these resemble closely both favus and herpes tonsurans, and on microscopic examination, the fungus can be detected. In fact, this eruption is owing to the fungus, which finds a fit receptacle in the macerated cuticle. As to the mode in which the fungus arrives at this cuticle, there can be no doubt that in many cases it is brought on the fomenting cloths. In other cases, its presence cannot be so easily accounted for.

Sometimes favus and herpes tonsurans occur at different parts on the same individual, and Hebra evidently inclines to the opinion, though he will not absolutely decide the point, that the two diseases are owing to the same fungus, at different periods of its growth.

The *treatment* of ringworm is said to be very successful. No internal remedies are given, but the cuticle and the hair are both removed, by applying a mixture of caustic potash and lard. This ointment is rubbed for ten minutes, night and morning, on the head, during four to six days; a flannel cap is worn during the days the rubbing is carried on, and for several days after, until the cuticle is detached, and the normal-colored skin is seen below. The head is washed with warm water, and the cure is complete. The entire time occupied is about twelve days. If the disease be on the scalp, the hairs must be pulled out.—*British and Foreign Medico-Chir. Review.*

